# In Vitro Evaluation of Flexural Strength, Impact Strength, and Surface Microhardness of *Vaccinium macrocarpon* Reinforced Polymethyl Methacrylate Denture Base Resin

**DOI:** 10.1002/cre2.70145

**Published:** 2025-05-19

**Authors:** Anitha Kuttae Viswanathan, Rajkumar Krishnan

**Affiliations:** ^1^ Department of Prosthodontics SRM Dental College Chennai Tamil Nadu India; ^2^ Department of Oral Pathology SRM Dental College Chennai Tamil Nadu India

**Keywords:** cranberry, denture base resin, polymethyl methacrylate

## Abstract

**Objective:**

The antimicrobial efficacy of cranberry (CA) against oral infections was well evidenced. Influence of cranberry on the mechanical properties of heat‐activated polymethyl methacrylate (HA‐PMMA) denture base resin (DBR) is unexplored till date and is significant for a better understanding of the reinforcement. This study aimed to evaluate the flexural strength (FS), impact strength (IS), and surface microhardness (VHN) of heat‐cure PMMA DBR reinforced with varying concentrations of *Vaccinium macrocarpon* (cranberry) extract.

**Material and Methods:**

A total of 150 samples were categorized into five groups (*n* = 10) by weight percentage of 0, 0.5, 1.0, 1.5, and 2.0 cranberry extract added into HA‐PMMA polymer after the performance of antimicrobial efficacy testing of CA. Three‐point bending test for FT, Izod impact testing for IS, and Vickers microhardness test were performed. Fractured sample surface was characterized by a high‐resolution scanning electron microscope (HR‐SEM). Raw data were statistically analyzed with one‐way ANOVA and post hoc Bonferroni test.

**Results:**

A significant improvement in flexural strength of 76.88 ± 0.73 MPa, impact strength of 6.66 ± 0.24 kJ/m^2^, and microhardness of 18.44 ± 0.27 kg/mm^2^ was observed at 2 wt.% (*p* < 0.0001). Fractured surface topography showed dispersion of cranberry particles as a thin fibrous band intermeshed within resin matrix.

**Conclusions:**

Addition of up to 2 wt.% cranberry improved the FS, IS, and VHN on comparison to 0 wt.% control HA‐PMMA.

## 1 Introduction

Removable dentures are mostly fabricated from polymethyl methacrylate (PMMA) denture base resins (DBRs) (de Oliveira Limírio et al. 2022). Heat‐activated PMMA material has proven many advantages such as easy handling with no requirement of expensive processing equipment, minimal cost, appealing esthetics, and simple to modify or repair (Alqutaibi et al. 2023). Limitations in mechanical properties, thermal conduction and surface characteristics with long‐term usage made the material suboptimal (Zafar 2020). Formation of denture biofilms attracting more pathogens, loss of surface luster, and fracture of the prosthesis when subjected to masticatory loads or accidentally dropped are considered as consequences of denture wearing (Schmutzler et al. 2021; An et al. 2021). To overcome this problem, continuous research is conducted over the years to reinforce PMMA with composite material. Studies have tried integration of synthetic drugs, herbal antimicrobials, fibers, nanoparticles of mineral oxides, ceramics to improve the clinical outcome (Hamid et al. 2019; Yerliyurt et al. 2023; Yadfout et al. 2023; Sivakumar et al. 2014; Abualsaud et al. 2021; Khan et al. 2022). Self‐cure PMMA (SC‐PMMA) reinforcement with 5% titanium oxide nanoparticles increased flexural strength (FS) and declined water sorption without impeding VHN and roughness (Abdelraouf et al. 2022). Incorporation of 0.5% of silver‐doped carbon nanotubes into SC‐PMMA evidenced improvement in FS, impact strength (IS), and VHN (Hamdy 2024). Limitations of PMMA‐based bone cement were overcome by the addition of 7 wt.% of halloysite nanotubes that increased compressive and flexural strength with reduced heat generation (Hamdy 2024). Each of these fortification substances has enhanced few properties over the others. Only when a material demonstrates satisfactory biological, mechanical and physical properties, it can be considered optimal for prosthesis making (Raszewski et al. 2021; McReynolds et al. 2023).

Prevalence of denture stomatitis (DS) poses a major challenge to prosthesis wearers (Hannah et al. 2017). It is represented by mild redness to hyperplastic papillary tissues in the denture‐bearing areas, causing great discomfort (Galvan et al. 2021). Daily proper denture hygiene measures need to be followed to limit the infection. The surface of dentures harbors microorganisms, and if proper oral hygiene is not maintained, these microbes spread to the systemic circulation, potentially leading to serious conditions such as aspiration pneumonia, systemic fungal infections, and other complications (Kulak‐Ozkan et al. 2002; Naik and Pai 2011; Lyu et al. 2016). For many years medicines such as nystatin, azole derivatives, amphotericin B, antiseptics, disinfectants, and other nonpharmacological measures in the form of physical brushing, photodynamic therapy, lasers, microwave sterilization and cleansing tablets are followed (Koray et al. 2005; Contaldo et al. 2023; Abuhajar et al. 2023; Schoeffel et al. 2023; Ribeiro et al. 2019; Vila‐Nova et al. 2023; Kaur et al. 2024; Santos Sousa et al. 2021; Pellizzaro et al. 2012; Anitha and Rajkumar 2023). Antimicrobial drugs have several drawbacks, including numerous side effects, recurrence of infection after treatment ends, interactions with other medications commonly used by the elderly, the development of antimicrobial resistance, higher costs, the necessity for strict patient adherence, and disruption of the normal flora (Rodrigues et al. 2019; Kubizna et al. 2024; Zhao et al. 2020). Understanding its management has led to a greater reliance on phytomedicines as an alternative to allopathic medications.

American Cranberries (CA) scientifically known as *Vaccinium macrocarpon* is a fruit widely consumed in different forms as fresh/dry fruit, juice, sauce, jelly, supplements, tea, and extract powders owing to its many health benefits (Blumberg et al. 2013). It has proven efficacy as anti‐inflammatory, anti‐infective, antioxidant, anticancer, immune booster, for improvement in cardiovascular, digestive, oral, and skin health (Nemzer et al. 2022; Côté et al. 2010). All these merits are attributed to the enriched bioactive phytochemicals it possesses (Bodet et al. 2008). In oral health care, cranberries have demonstrated limited dental plaque formation, reduction of periodontal inflammation, antibiotic, antifungal effect against oral pathogens, decrease in halitosis, enhancement of wound healing, oral cancer protection, increase in salivary production, and overall, for the maintenance of good oral hygiene as a mouthrinse. (Bonifait and Grenier 2010; Labrecque 2006; Steinberg 2004; Im et al. 2017). To date, only a few studies have been done on the incorporation of cranberry extract into denture‐making material in self‐cure PMMA denture polymer (Anitha and Rajkumar 2022, 2024).

Stomatitis is not the only complication of denture wearing, but chances of prosthesis fracture, breakage on sudden impact, and loss of surface characteristics are other major problems reported (International Organization for Standardization 2013). The deterioration of these properties compromises the durability of the prosthesis, diminishes patient comfort, and adversely affects the overall clinical outcome. In certain cases, it might be necessary to reconstruct the dentures. Despite the well‐established antimicrobial benefits of *V. macrocarpon* (cranberry) extract in oral health care, evidence regarding its effect on the mechanical performance of DBRs remains limited. Considering persistent clinical challenges such as denture stomatitis, surface degradation, and prosthesis fracture, the present study sought to investigate whether the incorporation of cranberry extract into heat‐cure PMMA DBR could enhance mechanical properties such as flexural strength, impact strength, and surface microhardness without compromising its structural integrity. This study aimed to evaluate the flexural strength, impact strength, and surface microhardness of heat‐cure PMMA DBR reinforced with varying concentrations of *V. macrocarpon* (cranberry) extract. The null hypothesis of the study stated that there would be no differences in the mechanical properties between cranberry‐reinforced and ‐unreinforced DBR.

## 2 Materials and Methods

This study was commenced after approval from the Institutional Review Board (XXXX/IRB/2018/PhD/No.101). No informed consent or ethical clearance for the protection of human subjects and animals in research was required, as the research design was an in vitro study. Preliminary antimicrobial efficacy assessment of cranberry extract was analyzed by performing level of inhibition zone (ZOI) test, calculation of lowest inhibitory dose (MIC), least bactericidal (MBC), and fungicidal dosages (MFC), and 3‐(4,5‐Dimethylthiazol‐2‐yl)‐2,5‐Di phenyl tetrazolium Bromide assay cyto‐toxicity (MTT) test. Quantitative and qualitative bioactive phytochemical analysis was done with chromatographic and spectrometric methods. On CA dosage optimization, a total of 150 samples (*n* = 10) were prepared for various mechanical property testing with an alpha error/probability set at ≤ 0.05. All the study samples were categorized into five groups (A–E) based on the varying proportion of cranberry extract it contained. Extract of cranberry rich in bioactive phytoconstituents was obtained following a similar protocol adhered in the previous study. Group A served as the control as there was zero percent addition of cranberry followed by incorporation of 0.5, 1.0, 1.5, and 2.0 dry wt. % in Groups B–E in order. The samples were fabricated according to the standardized guidelines as shown in Table 1.

**Table 1:** Mechanical property tests done in the study.

Tests done	*n*	Standards	Specimen dimension (mm)
**Flexural strength (FS)**	10	ISO 20795‐1:2013	64 × 10 × 3.3 mm ± 0.01 (rectangular bar)
**Impact strength (IS)** **Vickers Microhardness (VHN)**	10 10	ASTM D256‐10 ADA No. 12	63.5 × 12.7 × 4 mm ± 0.01 with R1 scale/hammer (rectangular bar) 65 × 10 × 2.5 mm ± 0.01 (rectangular bar)

### 2.1 Antimicrobial Efficacy Assessment of Cranberry Extract

The antimicrobial efficacy of cranberry extract was evaluated using the agar well diffusion method against *Streptococcus mutans*, *Staphylococcus aureus*, and *Candida albicans*, with standardized cultures (0.5 McFarland) inoculated on respective agar media. Zones of inhibition were measured after 24‐h incubation at 37°C. MIC values were determined using serial dilutions (5 mg/mL to 0.00005 mg/mL) in microtiter plates, with microbial growth monitored by optical density at 630 nm. The MIC was defined as the lowest concentration with ≥ 50% inhibition, while MBC/MFC values were identified from subcultures showing 99.9% microbial kill on agar. Cytotoxicity was assessed via MTT assay using Vero 76 cells cultured in Dulbecco's Modified Eagle Medium (DMEM). After treatment with extract dilutions, MTT dye was added, and formazan formation was quantified by measuring absorbance at 570 nm using a microplate reader, determining cell viability and safety of the extracts. Advanced phytochemical profiling was performed using high‐resolution liquid chromatography and mass spectrometry (LC‐MS) (410 Prostar Binary LC with 500 MS IT PDA detectors, IIT Mumbai) to identify bioactive compounds in cranberry extract. Mass spectra were analyzed using m/z Cloud and validated via ChemSpider for structural confirmation of the phytonutrients.

### 2.2 Specimen Preparation

An electronic weighing balance (Mettler Toledo) was used for the measurement of CA extract and HA‐PMMA polymer. To be certain of uniform homogenity of both the powders, it was subjected to planetary ball milling for 30 min at 300 rpm (PM 100 C; Retsch GmbH). The blended CA‐PMMA was then used for final processing of the specimen with “lost‐wax technique.” To affirm correct specimen dimension, in this study a three dimensionally (3D) printable wax based resin (Siraya Tech 3D) was used for making the patterns. The specimen's Standard Tessellation Language (STL) file was generated using computer‐aided design software, specifically Autodesk 123D Design. Use of Digital light processing (DLP) 3D printer (Anycubic Photon Ultra DLP) at specifications of 0‐degree direction, thickness of 30 μm and layer curing at 6.4 s was adhered. On finalization of digital printing, the patterns were cleansed with isopropanol in an ultrasonic bath. After it was post‐polymerized in a UV light curing unit for 30 min (Delta Blue Lux light curing unit). 3D printed patterns were then flasked, de‐waxed, packed, and heat cured by long polymerization cycle. Cured samples were finished with acrylic trimmers, sandpapered with different grits of 100, 150, 220, wet grinding on emery sheet of 600, 1000, 1500 grit size, 2000 grit and dry polishing with diamond polishing paste (Metco; 3‐4 Mic diamond paste) was done on a twin disc grinder/polisher at 600 rpm for 1 min (Metco Bainpol VTD). Final size of the samples was measured using a digital vernier calliper with an accuracy of 0.01 mm. Prepared samples were stored in distilled water at 37°C for 48 ± 2 h before testing.

### 2.3 Flexural Strength Testing

Flexural strength assessment using three‐point bend test on an universal testing machine (Autograph, AG‐IS 50 kN, Shimadzu Co., Japan) was performed after removal of the samples from distilled water without drying as per the ISO 20795‐1‐2013 guidelines (ASTM International 2010). The sample was horizontally secured with two vertical supports 50 mm apart. A central load was applied at a cross head speed of 5 mm/min until it fractured at which time the maximum fracture load was recorded. The flexural strengths of each specimen were calculated using the formula: FS = 3WL/2bd^2^, where FS is the flexural strength (in MPa), W is the maximum fracture load (in Newtons), L is the distance between the supports (50 mm), b is the specimen width (10 mm), and d is the specimen thickness (2.5 mm). The fractured specimen from each group was subjected to study the surface characteristics with scanning electron microscope (SEM) (Thermoscientific Apreo S; Model Quanta 250 FEG). For better perception, fractured surface was sputtered with gold (Quorum; Q150R ES) (Al‐Dwairi et al. 2023).

### 2.4 Impact Strength Testing

Izod impact strength tester was used for IS detection based on ASTM D256‐10 standards. A central V‐shaped notch 2.54 mm deep, at an angle of 45 degrees, and root radius of 0.25 mm was made. The specimen was secured vertically in the Izod testing machine (Presto Stantest Pvt. Ltd; IZC 222) with notch facing towards the side of pendulum. A pendulum load of 2.75 J was applied. The specimen fractured, while the pendulum was released to strike against the notch in the specimen. The amount of energy absorbed at the time of fracture was measured and recorded as joules (J) or kilojoules per square meter (kJ/m²). Cross‐sectional area of the specimen was measured as width x thickness. Impact strength was then calculated using the following formula:

ImpactStrength=Energyabsorbed/Cross−Sectionalarea(J/m)

### 2.5 Surface Microhardness Testing

According to American Dental Association Specification No. 12, the Vickers microhardness tester (Micro Vickers VH‑1MDX, Economet, Chennai Metco) measured surface hardness by using an indenter point in the shape of a square‐based pyramid (Chang et al. 2022). A load of 2.942 N at a 15‐s dwell time at room temperature was applied. For each specimen, three Vickers hardness indentations were made at different points along the specimen. The mean hardness was calculated and used for the statistical analysis.

### 2.6 Statistical Analysis

Descriptive and inferential statistics was analyzed with statistical software program (IBM SPSS Statistics for Windows, v21; IBM Corp). Mean and Standard deviation (SD) was used to summarize quantitative data. Intergroup comparison data between the five groups were done with One‐way ANOVA followed by Tukey's post‐hoc test for multiple pairwise comparison. Data was summarized with appropriate bar charts. Throughout the study, a *p*‐value of < 0.05 was considered as a statistically significant difference.

## 3 Results

**Figure 1:**
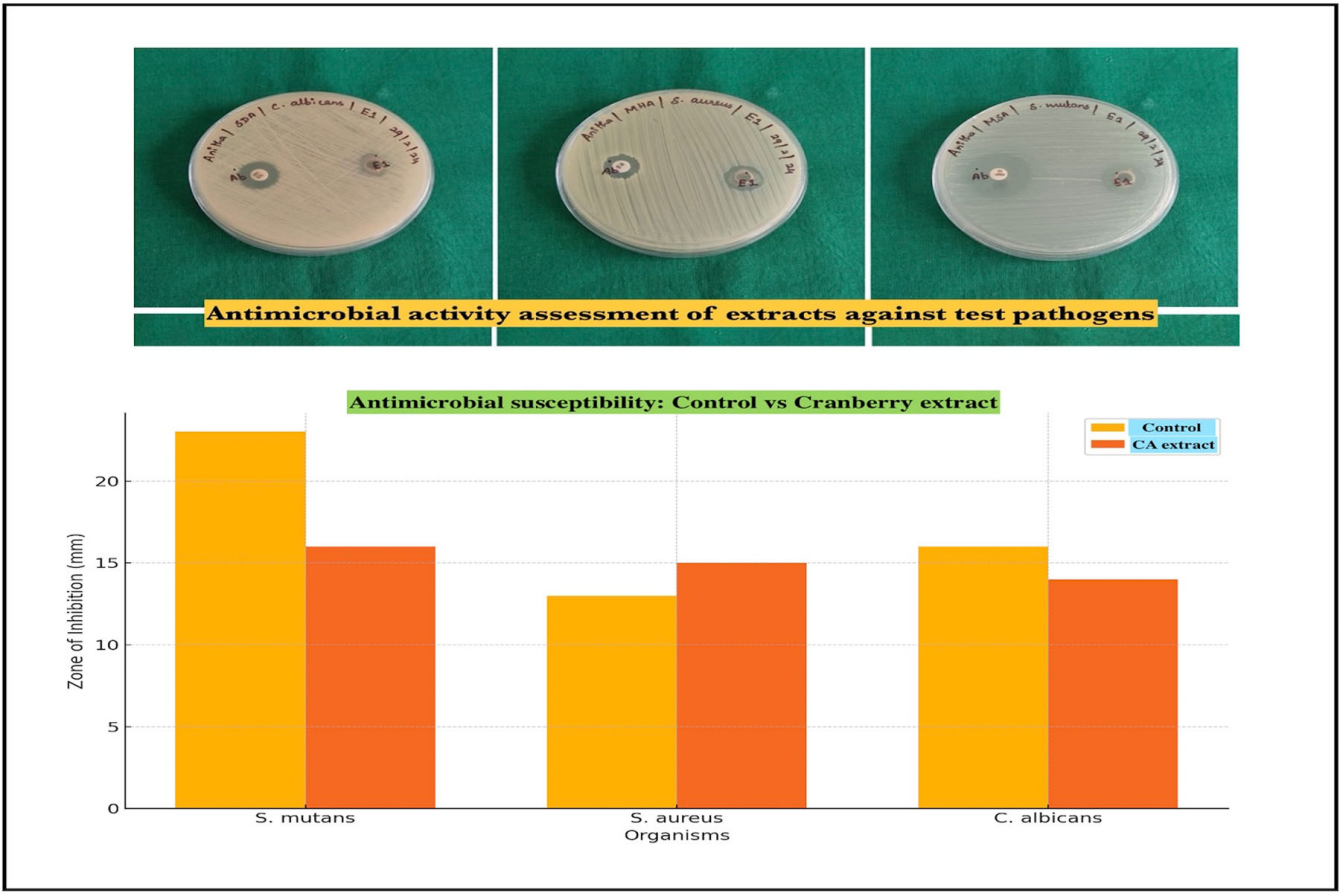
Antimicrobial susceptibility of cranberry extract against synthetic drugs and comparison of inhibition zone.

**Figure 2:**
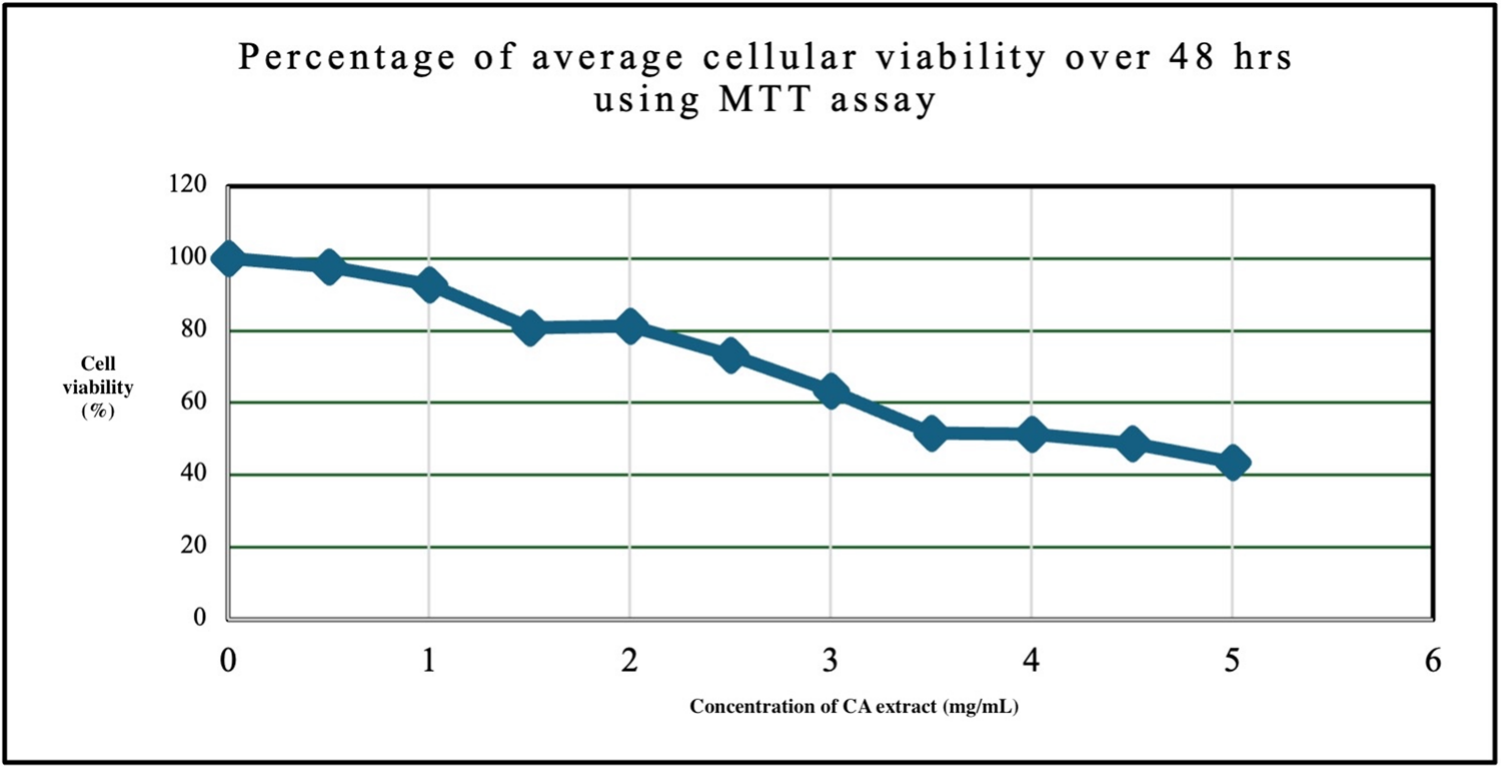
Assessment of in vitro cytotoxicity of cranberry extract with MTT assay.

**Figure 3:**
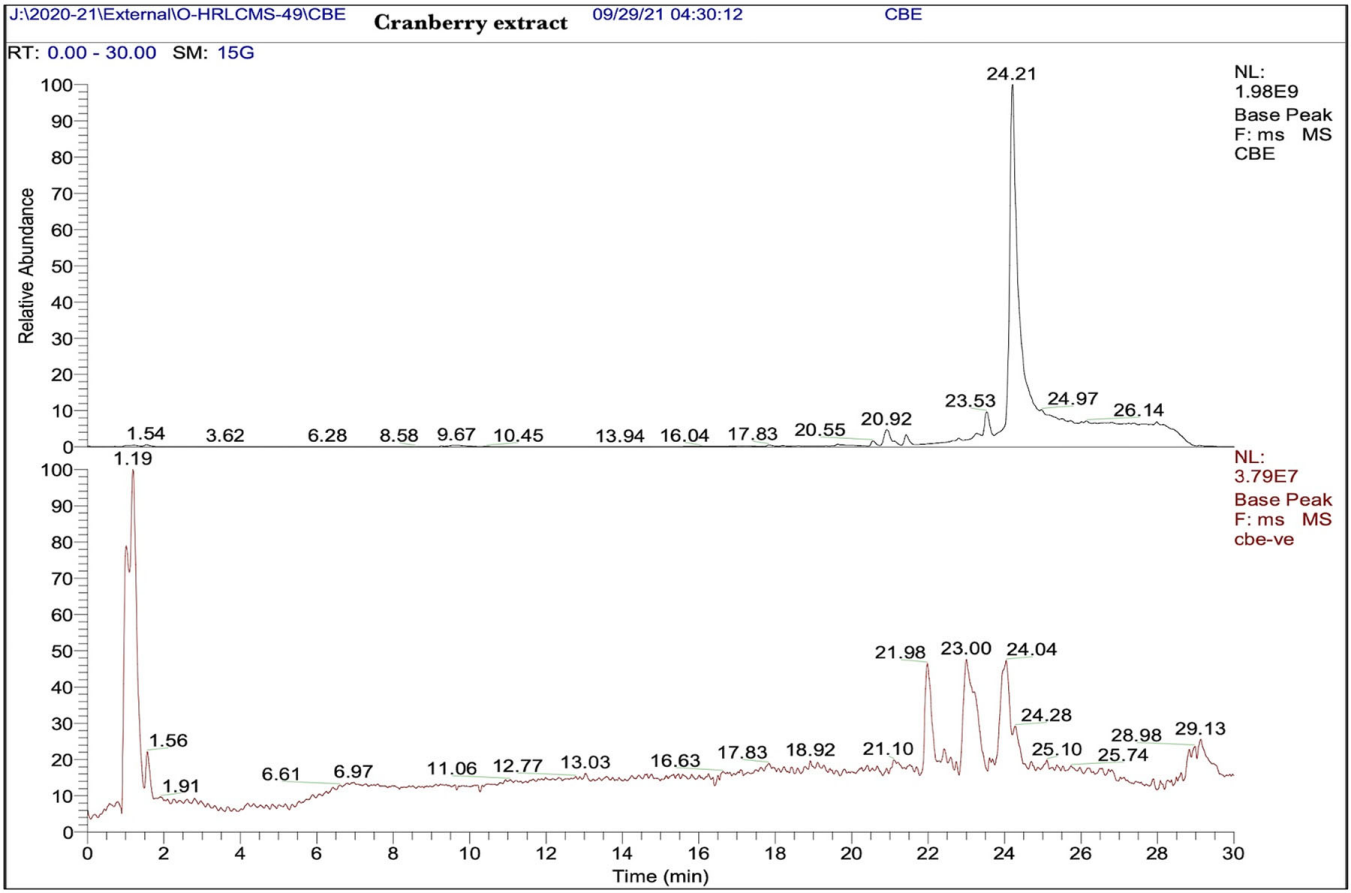
Chromatogram obtained after phytochemical analysis of cranberry extract with LC‐MS.

Antimicrobial tests results of cranberry extract showed its susceptibility against the three‐test pathogen, as illustrated in Figure 1. Higher *p*‐values than 0.05, indicated that observed differences between well‐established antimicrobial synthetic drugs and the cranberry plant extract were negligible and insignificant. Lowest concentration of cranberry extract, required to impede microbial growth was 0.5 mg/mL (10⁻²), achieving 95% inhibition for *S. aureus*, S. mutans, and 94% for *C. albicans*. At 10⁻³ (0.05 mg/mL), inhibition dropped to 40%, with no effectiveness at 10⁻⁴ and lower concentrations. MBC and MFC against all test pathogens showed a value of 5.0 mg/mL (10^−1^ dilution). In‐vitro cytotoxicity analysis demonstrated that the safe concentration of cranberry extract ranged up to 2 mg/mL. 80 to 100 percent of cells were viable at concentrations from 0 to 2.0 mg/mL. Beyond 2.0–5.0 mg/mL there was a significant decline in cell viability (Figure 2 **)**. LC‐MS analysis of cranberry extract revealed diverse phytoconstituents, including flavonoids such as quercetin‐3β‐d‐glucoside, phenolic acids, tannins, terpenoids, alkaloids, fatty acid derivatives (oleamide), and coumarins. Prominent peaks at 1.54–29.13 min indicated the presence of bioactive compounds with potential antioxidant, antimicrobial, and pharmaceutical applications (Figure 3).

The descriptive statistical data for flexural strength, impact strength, microhardness, and surface roughness were illustrated as mean ± SD in Table 2. Flexural strength was the highest with a value of 76.88 ± 0.73 MPa with 2% concentration of cranberry. Impact strength showed a significant increase from 5.44 ± 0.32 to 6.66 ± 0.24 kJ/m^2^ at 1% and 2%. Microhardness was elevated at 1.5% and 2% concentrations to 18.44 ± 0.27 kg/mm^2^ as compared against control which exhibited a value of 17.43 ± 0.51 kg/mm^2^. The mean values of flexural strength, impact strength, and microhardness sequentially increased on comparison to control group and were statistically significant (*p* = 0.0001). The significant *F* and *p*‐values (*p* < 0.05) indicated that there were statistically significant differences between the groups for all measured properties.

**Table 2:** Descriptive statistics of specimen and properties.

		Mean	SD	95% Confidence Interval for Mean		*F*	*p* value
				Lower Bound	Upper Bound		
Flexural strength (MPa)	Control	73.7710	0.80685	73.1938	74.3482	38.98	0.0001
	0.5%	73.2160	0.78218	72.6565	73.7755		
	1%	73.9710	0.79761	73.4004	74.5416		
	1.5%	74.4040	0.42167	74.1024	74.7056		
	2%	76.8880	0.73607	76.3614	77.4146		
Impact strength (kJ/m^2^)	Control	1.6079	0.71936	1.0933	2.1225	229.3	0.0001
	0.5%	1.3466	0.66504	0.8708	1.8223		
	1%	5.5378	0.44523	5.2193	5.8563		
	1.5%	5.4429	0.32064	5.2135	5.6723		
	2%	6.6684	0.24776	6.4912	6.8456		
Microhardness (kg/mm^2^)	Control	17.4320	0.51296	17.0650	17.7990	8.95	0.0001
	0.5%	17.7890	0.85068	17.1805	18.3975		
	1%	17.1300	0.74841	16.5946	17.6654		
	1.5%	18.4400	0.56999	18.0323	18.8477		
	2%	18.4400	0.27162	18.2457	18.6343		

One‐way ANOVA analysis followed by post hoc Bonferroni for estimated differences between and within the groups are shown in Tables 3 and 4. In all the mechanical properties tested, a highly significant difference was observed in the mean values between and within the groups (*p* < 0.0001). Overall, the 2% concentration evidenced to have significantly higher values in most parameters, indicating a strong impact of this concentration level on the material properties studied.

**Table 3:** ANOVA analysis of various properties between and within groups.

		Sum of Squares	df	Mean Square	*F*	*p* value
Flexural strength	Between Groups	81.592	4	20.398	38.948	0.0001
	Within Groups	23.567	45	0.524		
	Total	105.159	49			
Impact strength	Between Groups	242.573	4	60.643	229.330	0.0001
	Within Groups	11.900	45	0.264		
	Total	254.473	49			
Microhardness	Between Groups	13.930	4	3.482	8.950	0.0001
	Within Groups	17.510	45	0.389		
	Total	31.440	49			

**Table 4:** Bonferroni multiple comparisons test of samples within the groups.

Parameter	Group comparing	Group compared	Mean Difference	*p* value	95% Confidence Interval	
					Lower Bound	Upper Bound
Flexural strength	Control	0.5% 1% 1.5% 2%	0.55500 −0.20000 −0.63300 −3.11700*	0.933 1.000 0.567 0.000	−0.4004 −1.1554 −1.5884 −4.0724	1.5104 0.7554 0.3224 −2.1616
	0.5%	1% 1.5% 2%	−0.75500 −1.18800* −3.67200*	0.242 0.006 0.000	−1.7104 −2.1434 −4.6274	0.2004 −0.2326 −2.7166
	1%	1.5% 2%	−0.43300 −2.91700*	1.000 0.000	−1.3884 −3.8724	0.5224 −1.9616
	1.5%	2%	−2.48400*	0.000	−3.4394	−1.5286
Impact Strength	Control	0.5%	0.26130	1.000	−0.4176	0.9402
		1%	−3.92993*	0.000	−4.6088	−3.2510
		1.5%	−3.83503*	0.000	−4.5139	−3.1561
		2%	−5.06054*	0.000	−5.7394	−4.3816
	0.5%	1% 1.5% 2%	−4.19123* −4.09633* −5.32184*	0.000 0.000 0.000	−4.8701 −4.7752 −6.0007	−3.5123 −3.4174 −4.6429
	1%	1.5% 2%	0.09490 −1.13061*	1.000 0.000	−0.5840 −1.8095	0.7738 −0.4517
	1.5%	2%	−1.22551*	0.000	−1.9044	−0.5466
Microhardness	Control	0.5%	−0.35700	1.000	−1.1805	0.4665
		1%	0.30200	1.000	−0.5215	1.1255
		1.5%	−1.00800*	0.008	−1.8315	−0.1845
		2%	−1.00800*	0.008	−1.8315	−0.1845
	0.5%	1% 1.5% 2%	0.65900 −0.65100 −0.65100	0.225 0.241 0.241	−0.1645 −1.4745 −1.4745	1.4825 0.1725 0.1725
	1%	1.5% 2%	−1.31000* −1.31000*	0.000 0.000	−2.1335 −2.1335	−0.4865 −0.4865
	1.5%	2%	0.00000	1.000	−0.8235	0.8235

**Figure 4:**
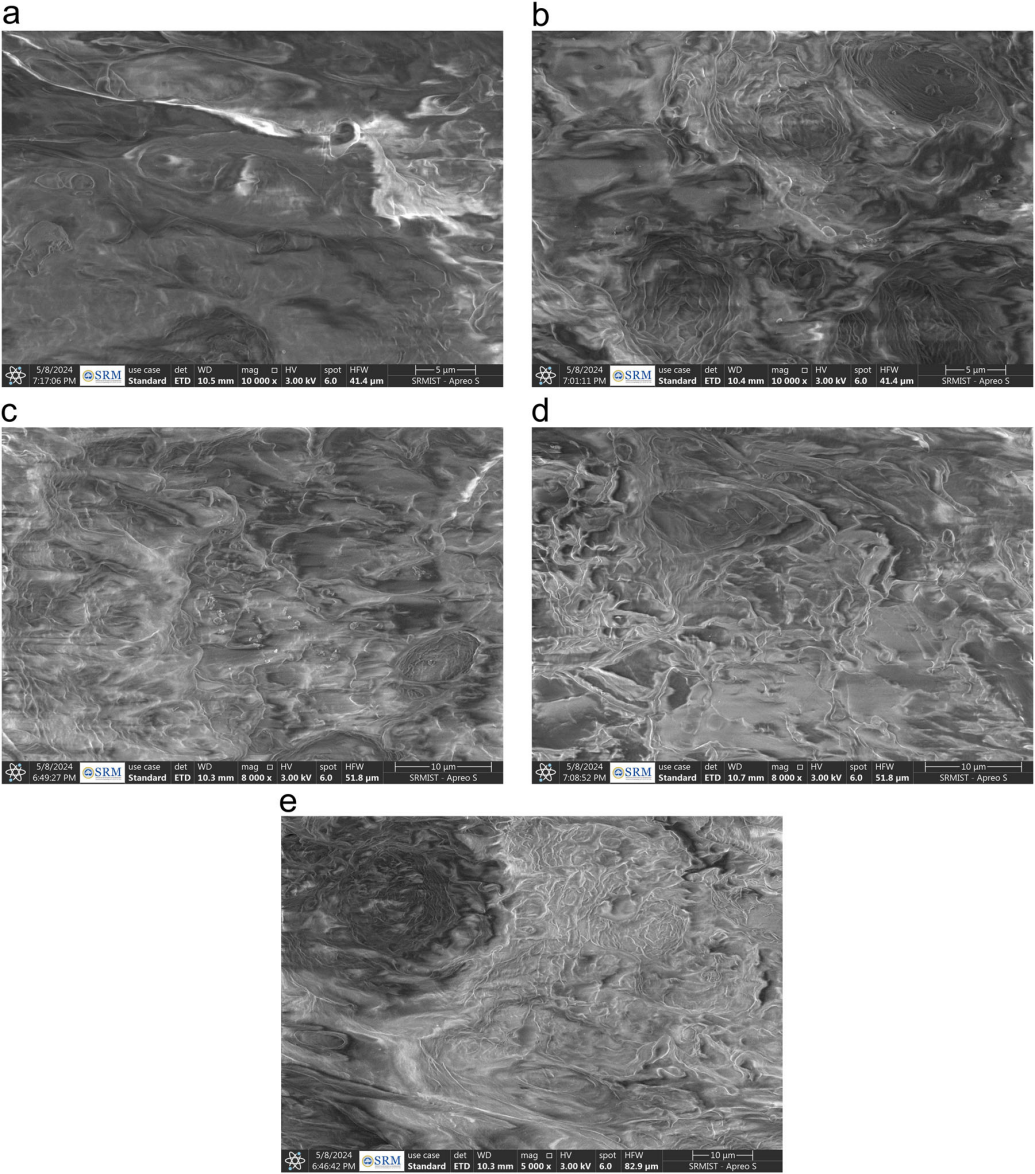
(a) SEM image of control sample. (b) SEM image of 0.5% CA sample. (c) SEM image of 1.0% CA sample. (d) SEM image of 1.5% CA sample. (e) SEM image of 2.0% CA sample.

Scanning electron microscopic analysis of the fractured sample evidenced spatial distribution of cranberry extract across the polymer matrix of DBR in all the reinforced groups. The cranberry extract particles appeared irregular in shape in the form of fibrous threads that was intermeshed within the resin matrix. There was a decline in porosity observed with the addition of cranberry extract as compared against control group (Figure 4a–e).

## 4 Discussion

From results of the present investigation, it was perceived that there was difference between all the groups when compared against the control samples. There was improvement in flexural strength, impact strength and microhardness after cranberry extract reinforcement into DBR. As the differences were statistically significant (*p* < 0.05), the null hypothesis was rejected. Flexural strength increased with the rise in CA extract concentration. The probable reason for this enhancement can be attributed to the phytochemical compounds present in cranberries. It is rich in polyphenols, flavonoids, which serve as natural antioxidants to stabilize the polymer matrix by limiting oxidative degradation (Côté et al. 2010). Additionally, this reduction in oxidative breakdown of the polymer chains, enhanced the overall integrity of the reinforced material, contributing to higher flexural strength was supported by Bodet et al. (2008). Presence of polyphenols and bioactive compounds in cranberry has a potential to cross‐link with the PMMA structure. Chang et al. stated the hydroxyl (–OH) groups in polyphenols bind to the carbonyl groups (C = O) in PMMA to form hydrogen bonds. The oxygen in the ester functional group of –COOCH3 present in the resin acts as hydrogen bond acceptors to facilitate this resin–CA extract chemical bonding (Golovinskaia and Wang 2021). Cranberry also contains more fibrous constituents that aid to resist more forces exerted on the samples by increased adhesion between the resin–fiber matrix (Vallittu 1999). This was supported in previous studies done by Yerliyurt et al. Vallittu, Narva, and Sundar showed improved flexural strength with fiber reinforcement (Narva et al. 2005; Sundar 2024; Balawejder et al. 2023).

By protecting the integrity of the polymer chains, the reinforced material's ability to absorb and dissipate energy during application of sudden high force, increased the impact strength. Presence of natural cross‐linking agents created a more interconnected and robust network within the material, added to the resistance of impact forces applied in the testing (Philip and Walsh 2019). The bioactive compounds in cranberry helped in matrix toughening by crack bridging, deflection and energy dissipation (Srivastava et al. 2023). With more proportion of cranberry, the reinforced material absorbed more energy before failing, thus increasing the impact strength. Uniform distribution of extract particles contributed to even distribution of stress during impact and made it to better resist localized failure (Tipton et al. 2013).

Addition of cranberry increased the microhardness of the PMMA material. Balewejder et al. in his in vitro analysis perceived the antioxidants present in the extract, scavenge the free radicals during the curing of polymer, and increase the resin stability. It reduced the chance of chain scission or completion of polymerization process (Philip and Walsh 2019). This enabled the formation of uniform, dense polymer network which was harder and more resistant to surface indentations (Labrecque 2006; Sundar 2024). A combination of chemical interaction, enhanced polymer matrix, and the reinforcing effect of cranberry extract particles improved the flexural strength, impact strength, and surface microhardness of the reinforced material.

The SEM images of fractured specimens after flexural strength testing could be associated to the improvement in FS, IS, and VHN values. Uniform distribution of the particles was noticed intermeshing with the core resin matrix in the form of longitudinal thin fibrous bands. Addition of cranberry extract decreased the number of voids or porosities in the resin. It was attributed to the enhancement in polymerization efficiency, presence of filler particles that bridged the micro gaps, improved chemical bond between polyphenols in cranberries and resin matrix, and reduction in surface tension that decreased the formation of air bubble entrapment and led to fewer porosities in the composite material (Rahman et al. 2021).

The versatile properties of cranberries are well demonstrated in the control of many oral diseases. This investigation has shown that addition of cranberry extract into denture‐making material has a significant impact on its mechanical properties. It is important to deduce the right concentration of cranberry with balanced properties for a better clinical outcome. In this study, 2% cranberry concentration showed the highest flexural strength, impact strength, and surface microhardness of the reinforced material. This inference must be understood within its limitations. The study was an in vitro analysis done with samples based on standardized guidelines. These samples differ in design, size, and shape when compared to dental prosthesis. The concentration of cranberry extract was limited to a maximum of 2 wt.% in the present study, as higher concentrations demonstrated a reduction in cell viability below acceptable thresholds, as observed in cytotoxicity assays. This restriction was necessary to ensure biocompatibility of the modified denture base material for potential intraoral applications. The present study did not evaluate the long‐term stability assessment of the cranberry‐reinforced PMMA resin. While short‐term in vitro results were encouraging, the potential effects of aging, storage conditions, and prolonged exposure to the oral environment remain a key area for future investigation. The potential for discoloration of PMMA due to the natural pigments present in cranberry extract has been recognized as a possible concern. Therefore, further studies focusing on the color stability of cranberry‐reinforced PMMA are recommended to ensure long‐term esthetic acceptability of the prosthesis.

In addition to the parameters assessed in the present study, future research should focus on evaluating other critical mechanical properties such as tensile strength, fatigue resistance, and wear behavior. Physical properties including water sorption, solubility, and dimensional stability, as well as thermal properties like thermal conductivity and glass transition temperature, also warrant investigation. A comprehensive assessment of these attributes is essential to determine the long‐term clinical performance and reliability of cranberry‐reinforced PMMA denture base materials. Reinforcement in heat‐activated PMMA DBR was tested. Further studies are needed to assess cranberry's effects on newly available 3D‐printed or injectable resins. Additionally, if the testing is done in clinically simulated environments, a better perception of the biomaterial can be gained.

## 5 Conclusion

The incorporation of cranberry extract into the polymer of heat‐activated PMMA DBR improved the flexural strength, impact strength, and surface microhardness. A sequential enhancement in all the three properties was appreciated from 0.5 to 2 wt.% while compared to control (0 wt.%) group. Highest mean values was observed at 2 wt.% and was considered as optimal for addition into the resin for improved reinforcement.

## Author Contributions

**Kuttae Viswanathan Anitha:** conceptualization, methodology, project administration, validation, original draft writing, editing, reviewing. **Rajkumar Krishnan:** conceptualization, methodology, project administration, validation, original draft writing, editing, reviewing.

## Conflicts of Interest

The authors declare no conflicts of interest.

## Data Availability

The data that support the findings of this study are available from the corresponding author upon reasonable request. The data from this study will be made available upon request to the author.
